# Development of cysteine-doped MnO_2_ quantum dots for spectrofluorimetric estimation of copper: applications in different matrices

**DOI:** 10.1007/s00216-023-04827-z

**Published:** 2023-07-11

**Authors:** Baher I. Salman, Ahmed I. Hassan, Roshdy E. Saraya, Adel Ehab Ibrahim, Bassam Shaaban Mohammed, Hany A. Batakoushy, Sami El Deeb, Yasser F. Hassan

**Affiliations:** 1 Pharmaceutical Analytical Chemistry Department, Faculty of Pharmacy, Al-Azhar University, Assiut Branch, Assiut, 71524 Egypt; 2 Pharmaceutical Analytical Chemistry Department, Faculty of Pharmacy, Port Said University, Port Said, 42511 Egypt; 3 Natural and Medical Sciences Research Center, University of Nizwa, Birkat Al Mauz, P.O. Box 33, Nizwa, 616 Sultanate of Oman; 4 Department of Pharmaceutical Analytical Chemistry, Faculty of Pharmacy, Menoufia University, Shibin-Elkom, 32511 Egypt; 5 Institute of Medicinal and Pharmaceutical Chemistry, Technische Universitaet Braunschweig, 38106 Braunschweig, Germany

**Keywords:** Cys@MnO_2_ QDs, Copper, Food samples, Fluorescence spectroscopy, Hair samples

## Abstract

Copper (Cu) plays a role in maintaining healthy nerve cells and the immune system. Osteoporosis is a high-risk factor for Cu deficiency. In the proposed research, unique green, fluorescent cysteine-doped MnO_2_ quantum dots (Cys@MnO_2_ QDs) were synthesized and assessed for the determination of Cu in different food and hair samples. The developed quantum dots were synthesized with the help of cysteine using a straightforward ultrasonic approach to create 3D fluorescent Cys@MnO_2_ QDs. The resulting QDs’ morphological and optical characteristics were carefully characterized. By adding Cu ions, the intensity of fluorescence for the produced Cys@MnO_2_ QDs was found to be dramatically reduced. Additionally, the applicability of Cys@MnO_2_ QDs as a new luminous nanoprobe was found to be strengthened by the quenching effect grounded on the Cu–S bonding. The concentrations of Cu^2+^ ions were estimated within the range of 0.06 to 7.00 µg mL^−1^, with limit of quantitation equal to 33.33 ng mL^−1^ and detection limit equal to 10.97 ng mL^−1^. The Cys@MnO_2_ QD technique was applied successfully for the quantification of Cu in a variety of foods, including chicken meat, turkey, and tinned fish, as well as in human hair samples. The chance that this novel technique could be a useful tool for figuring out the amount of cysteine in bio-samples is increased by the sensing system’s remarkable advantages, which include being rapid, simple, and economical.

**Graphical abstract:**

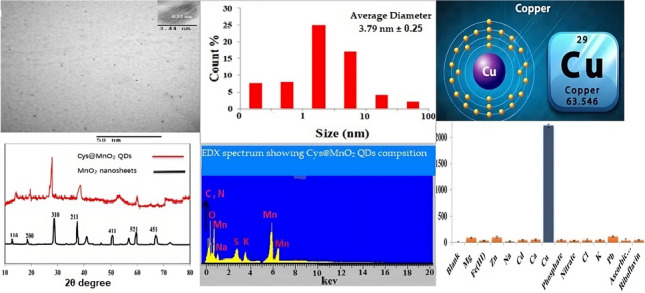

**Supplementary Information:**

The online version contains supplementary material available at 10.1007/s00216-023-04827-z.

## Introduction

Copper is very essential for organisms; it plays a critical role in numerous biochemical processes and is necessary for enzyme actions [1]. While most vegetarian and botanical nutrition sources are high in copper, the human body only stores about 50–120 mg and excretes excess copper through hepatic excretion. Prolonged exposure to elevated concentrations of copper via polluted diets and water sources leads to hepatitis, leukemia, diabetes, renal failure, tumor diseases, brain damage, and occasionally, copper toxicity, which is fatal despite treatment. Certain hereditary diseases, such as Wilson’s and Menkes syndromes, can additionally arise from copper poisoning [2, 3].

Various methods were established for copper detection and quantification such as atomic absorption spectrometry, inductively coupled plasma with mass detection spectrometry (ICP/MS), spectrofluorimetry, electrochemical analysis, spectrophotometry, and nanomaterials [4–8]. Among the reported methods, those using biocompatible nanomaterials and quantum dots were found to be the most advantageous in terms of selectivity, sensitivity, low cost, and sustainability [9, 10]. Different carbon dot methods were utilized for the estimation of copper [11–15]. The main disadvantage of the reported use of carbon dots is its lack of selectivity compared with the presented method. Although copper ions were also determined via numerous transition metal nanostructures, such as CdSe/CdS, ZnSe, CdTe, and ZnS [16–18], MnO_2_ is one of the ideal nominees among them due to several advantages. Their high luminescence quantum yield and low cost, in terms of manpower and ecological costs, are some of these merits. Other desirable factors include their enzymatic-like action, good bio-compatibility, beneficial therapeutic properties, broad application index, and ultrahigh theoretical specific capacitance [19]. Moreover, because of its outstanding reducing capacities and biocompatible properties, cysteine was frequently employed as a ligand for the construction of several nano-structures that were used in different biomedical applications [20, 21]. Cysteine functions as a dual-purpose ligand that is adsorbed on the surface of MnO_2_ nanosheets. Depending on the strength of the intramolecular hydrogen bonds, cysteine then inserts within the spaces of the adjacent layers of MnO_2_ inducing their enlargement, hence causing the massive MnO_2_ nanosheets to exfoliate into tiny nanosheets and break apart into MnO_2_ QDs [21].

The aim of this work is to establish a green, environmentally friendly, selective, and ultrasensitive method for the estimation of Cu using Cys@MnO_2_ QDs. The sensitivity of Cys@MnO_2_ QDs was also investigated for assessment of copper ions and their selectivity in the prevention of impurities in different kinds of food including chicken meat, turkey, and tinned fish, as well as in human hair samples.

## Experimental

### Instrumentation

A FS5-model spectrofluorometer from Edinburgh Instruments (Edinburgh, UK) was used to collect the data, and a 150-W xenon lamp source was used for excitation. A Shimadzu 1601PC UV–Vis spectrophotometer was used to obtain UV–Vis spectra. An FTIR instrument model Nicolet® iSTM10 spectrometer from ThermoFisher Scientific (MA, USA) was used to investigate the creation of functional groups. A transmission electron microscope (TEM) and scanning electron microscope (SEM) model JEM-100CXII from JEOL (Tokyo, Japan) were used to capture photographs of the surface morphology.

### Reagents and chemicals

KMnO_4_ was supplied by Oxford Laboratory Chemicals (Maharashtra, India). CuCl_2_ anhydrous and sodium oxalate were obtained from Isochem Chemicals (Vert-le-Petit, France). Cysteine and ascorbic acid were obtained from the United Company for Chemical and Medical Preparations (UCCMA, Cairo, Egypt). Food samples including chicken, meat, turkey, and canned fish were purchased from the local Egyptian market. Standard copper reference material was obtained from Labmix (Germany).

### Preparation of MnO_2_ nanosheets

With a small alteration, MnO_2_ nanosheets were prepared as previously described in literature [19]. An amount of 200 mg of KMnO_4_ was mixed in a solution at room temperature with ascorbic acid (0.05 g) in 200 mL of Briton Robinson (B.R. pH 7.0) buffer. The mixture was then placed in an ultrasonicator for 30 min, until the pink color of permanganate became a brown colloidal form. Subsequently, the obtained colloid was centrifuged for 30 min at 4000 rpm and the clear supernatant was decanted. The obtained brown precipitate was gathered, then washed three times using ultra-pure distilled water and absolute ethanol as well. Finally, the collected precipitate was dried at 60 °C for 3 h where MnO_2_ nanosheets were obtained.

### Preparation of Cys@MnO_2_ QD solution

For the creation of Cys@MnO_2_ QDs, MnO_2_ nanosheets were used as a precursor. In 50 mL of ultra-pure water, MnO_2_ nanosheets (50 mg) were combined with cysteine (50 mg/mL in water). The output mixture was then subjected to 30 min of sonication, after which the suspension underwent 30 min of 20,000 rpm centrifugation to separate any unexfoliated QDs. The supernatant solution was centrifuged again at 20,000 rpm for another 30 min after being sonicated for 30 more minutes. A 60 °C electric oven was used to dry the silt. The final product (weighing 100 mg) was dissolved in ultra-pure water (100 mL) and then homogenized using a sonicator for 30 min. The obtained homogeneous mixture was then filtered using a 0.2-µm membrane filter to obtain Cys@MnO_2_ QD solution (0.16 mg mL^−1^).

### Fluorescent sensing of copper

In a 5-mL volumetric flask, 0.8 mL of the Cys@MnO_2_ QD solution was combined with 1.0 mL of B.R. buffer (pH 4.5), and the final range (0.06–7.0 µg mL^−1^) was obtained after thorough mixing. Using ultrapure water, the solution was finished to specification. The spectral measurements were examined at 420 nm (ex 337 nm) after 12 min.

### Applications of the proposed method

The copper content of chicken meat, turkey, canned fish, and hair samples was extracted as previously reported [21–23]. Hair samples were collected from the scalp of an Egyptian human volunteer (a healthy male laboratory staff working at the Faculty of Pharmacy, Al-Azhar University) via a relatively easy and non-invasive procedure using forceps. Briefly, a 5.0-g weighed food sample was pulverized into homogenized masses. Then, 10 mL of concentrated HNO_3_ (65%, v/v) together with 15 mL of HClO_4_ (70–72%, v/v) was added to the homogenized samples. In order to produce a clear solution, the combination was digested in a microwave digestion for 7 min (400 W). A membrane filter made by Whatman® (GD/X PES) was used to filter the residue. Once it was cooled, 50 mL of ultrapure water was added to the solution. Afterwards, the advised method was applied to assess the copper content.

## Results and discussion

The importance of copper is in maintaining a healthy immune system and nerve cells. Therefore, the analysis of copper in food samples such as chicken meat, turkey, canned fish, and hair is very essential in improving food quality and public health improvement.

### Selection of surface modifier

Because of its good reducing and biocompatibility properties, cysteine is an excellent ligand for developing several nanostructures which were applied in biomedical areas [20, 21, 24]. In the existence of MnO_2_, cysteine can be oxidized into bisulfide cysteine, which in turn adsorbs physically on the exterior of MnO_2_ nanosheets, and then depending on the strong intra-molecular hydrogen bonds, it inserts into the gap between the adjacent MnO_2_ layers to create expansion circumstances, conducting the exfoliation of massive MnO_2_ nanosheets into tiny nanosheets and disintegration into MnO_2_ QDs. Eventually, it covalently bonds to the obtained QDs with thiol groups and makes the capped QD water soluble with carboxylic groups [20, 21, 24].

### Morphological characterization of Cys@MnO_2_ QDs

**Fig. 1:**
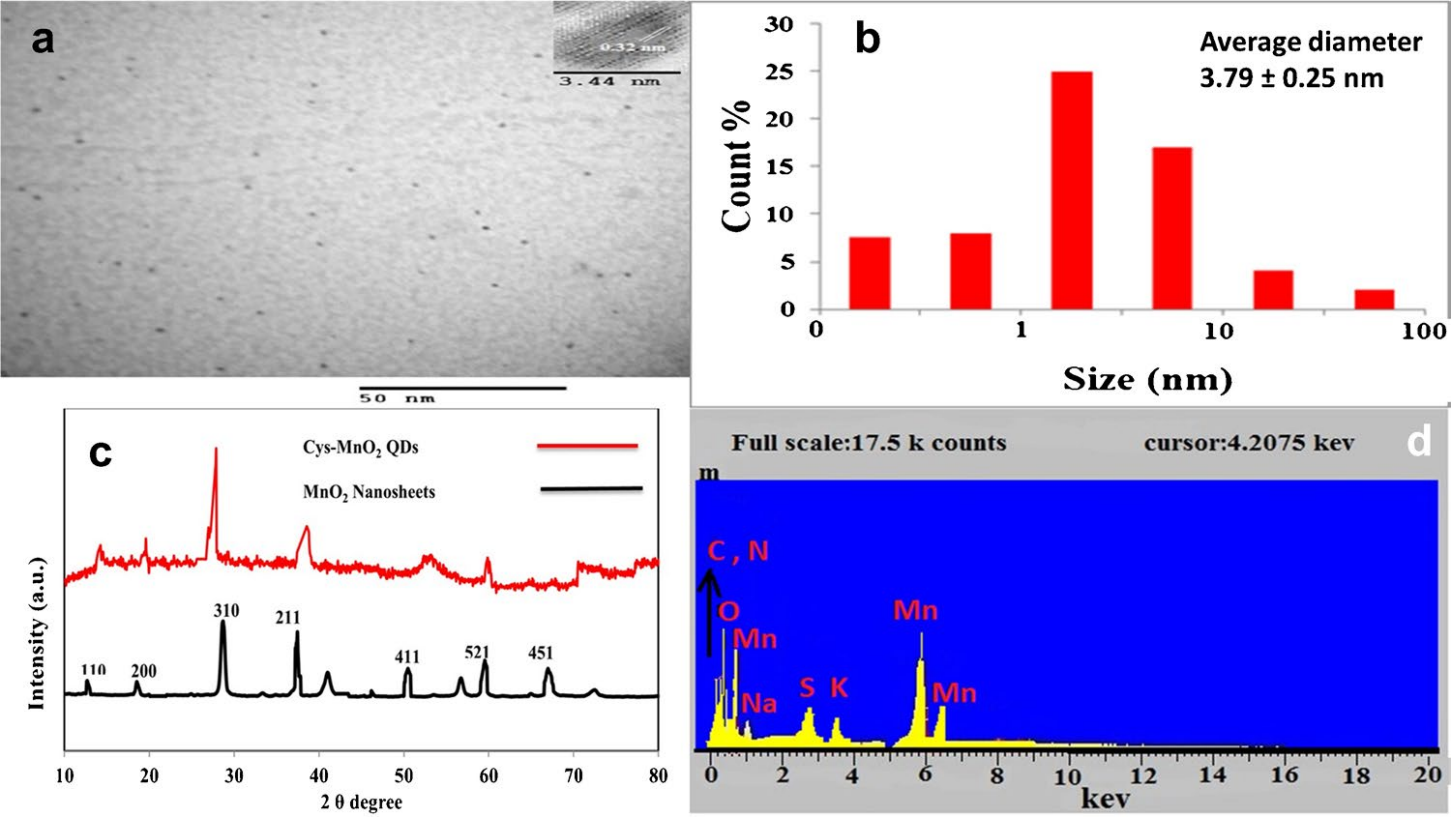
TEM images of Cys@MnO_2_QDs (**a**), DLS of Cys@MnO_2_QDs (**b**), PXRD (**c**), and EDX for Cys@MnO_2_QDs (**d**)

Figure 1a shows the surface morphology of the produced Cys@MnO_2_ QDs using TEM which were almost circular and uniform in size, ranging from 2.3 to 5.20 nm and having satisfactory dispersion. For more confirmation, their size measurement was carried out using DLS equipment (Fig. 1b). An average size of 3.79 ± 0.25 nm was obtained. This slight increase in size obtained by using DLS equipment than TEM might be attributed to the fact that the DLS technique provides the mean hydrodynamic diameter of the QDs’ core surrounded by a solvation layer that could be affected by the concentration and viscosity of the solution [21, 25, 26].

Besides, the surface morphology of the synthesized MnO_2_ nanosheets was investigated by SEM that demonstrated a compact and well-enveloped film surface with high morphological purity. Regarding Cys@MnO_2_ QDs, the SEM micrograph displays a 3D highly porous surface which offers a large surface area and hence represents the structural foundation for the high specific performance as shown in supplementary Figure S1.

Cys@MnO_2_ QDs were examined using PXRD as well as MnO_2_ nanosheets for their crystallinity (Fig. 1c). As shown, the diffraction peaks of the MnO_2_ crystal planes (110), (200), (310), (211), (411), (521), and (451) corresponded well with each other and with earlier results [19, 21, 27]. On comparing the two patterns, MnO_2_ nanosheets were discovered to have sharper and stronger peaks than those broader diffraction peaks for Cys@MnO_2_ QDs. This better crystallinity of the MnO_2_ nanosheets indicates the contact among MnO_2_ nanosheets and cysteine results [19, 21, 27].

Paul Scherrer's formula (Eq. 1) was used for calculating the average size of Cys@MnO_2_ QD nanoparticles for the (310) diffraction peak pattern [28].

1 $$L=K.\frac{\lambda }{\beta \mathrm{cos}\theta }$$

where *K* is Scherrer’s constant (*K* = 0.94), λ accounts for the wavelength of X-ray (*λ* = 1.542), *θ* is the corresponding diffraction angle (2*θ* = 27.88**°**), and *β* is the line broadening after subtracting the instrumental line broadening. Moreover, the obtained EDS spectrum for Cys@MnO_2_ QDs (Fig. 1d) confirmed the existence of Mn, C, N, O, and S in the sample which match those previously reported [20, 21].

The supplementary Figure S2 shows the FTIR spectra of pure cysteine and cyste@MnO_2_ QDs. Two peaks were resolved (3498 and 1624 cm^−1^) which correspond to the stretching vibrations of O–H and COO–, respectively. The well-resolved signal at 2618 cm^−1^ (the stretching vibrations of S–H), which is attributed to the rupture of the S–H bond and the creation of a new Mn–S bond, completely vanished when compared to the FTIR spectra of pure cysteine. This should be due to the bi-functional ligand character of cysteine, which could manage the thiol group to bind with Mn on the QDs’ exterior [29]. Meanwhile, the peak corresponding to the stretching vibration of –NH_2_ (2053 cm^−1^) that resulted from the basification of cysteine also disappeared during the synthesis of cyste@MnO_2_ QDs [29]. Additionally, other absorption peaks were detectable corresponding to Mn–S (644 cm^−1^), those related to the stretching collision of O–Mn–O for cyste@MnO_2_ (400 and 500 cm^−1^), and the surface –OH group of Mn–OH for Cys@MnO_2_ QDs (913 cm^−1^) [21]. It is worth noting that the assigned peak to the stretching vibration of the adsorbed molecular water (at 3500 cm^−1^) was maximized due to the stretching vibration of the –OH group of cysteine in the Cys@MnO2 QD product [19]. The scanned FTIR spectra for Cys@MnO_2_ QDs proved the surface capping of the synthesized QDs.

In addition, XPS for Cys@MnO_2_ QDs was performed for confirmation of the presence of sulfur and manganese elements (supplementary Figure S3a). Mn 2p spectra appeared at 643.9 and 655.8 eV corresponding to Mn 2P_3/2_ and Mn 2P_1/2_ as seen in supplementary Figure S3b. For S 2p, a characteristic peak was observed at 161.4 eV related to the Mn–S bond (supplementary Figure S3c).

### Optical characterization of Cys@MnO_2_ QDs

Cys@MnO_2_ QDs were found to have an absorption UV band within the region of 275–300 nm (Supplementary Figure S4). This band may be correlated to electron transition from the valence band to the conduction band. Meanwhile, the visible absorption band should originate principally from the d–d transition of Mn ions [27]. The photoluminescence emission peak of as-prepared net MnO_2_ QDs without the aid of cysteine is around 400 nm (red curve) while another of Cys@MnO_2_ QDs (blue curve) was enhanced to a certain extent and red-shifted and located at about *λ* = 420 nm (Supplementary Figure S4). The intensity enhancement and the red shifting of the emission peak of Cys@MnO_2_ QDs are attributed to the surface functional groups of Cys@MnO_2_ QDs, proving that cysteine promoted the exfoliation and shattering of MnO_2_ and then acts as a stabilizer to assist the metal–ligand charge transfer of Cys@MnO_2_ QDs which enables the QDs to possess a conservative micro-environment and in turn good photostability (Supplementary Figure S5) [30]**.**

The quantum yield (QY) of the Cys@MnO_2_ QDs was examined via quinine sulfate (QY = 0.55) [31] by reported methods [9, 10, 32] using Eq. 2.

2 $${\Phi }_{x}={\Phi }_{\mathrm{std}}({F}_{x}{A}_{\mathrm{std}}{\eta }_{x})/({F}_{\mathrm{std}}{A}_{x}{\eta }_{\mathrm{std}})$$

where the QY for Cys@MnO_2_ QDs was calculated to be 40.26%. This figure is considered high when compared to previously published reports [9, 16, 19].

### Study of Cu detection mechanism using Cys@MnO_2_ QDs

A distinct peak of Mn–S in the FTIR spectrum of Cys@MnO_2_ QDs confirms that the electron density and chemical surface processes have enhanced as a result of the inclusion of cysteine as a thiol-based capping agent onto the MnO_2_ QD surface. Due to their high capacity for complexation with S, Cu ions can take the place of surface Mn in the formation of Cu–S bonds with cysteine, which results in non-radiative decay and the dampening of the Cu concentration-dependent Cys@MnO_2_ QD fluorescence. As a control, reducing and oxidizing chemicals such ascorbic acid, glutathione, Na_2_S_2_O_3_, Fe^2+^, H_2_O_2_, and Fe^3+^ (at concentration 2.0 µg mL^−1^) were examined with the Cys@MnO_2_ QD solution in order to confirm this tactic and rule out the redox pathway.

Supplementary Figure S6 demonstrates that none of the listed species significantly reduced the fluorescence of Cys@MnO_2_ QDs, proving that the redox mechanism is not to blame for the dampening of the fluorescence response of the interaction between the Cys@MnO_2_ QDs and Cu. To reinforce the proposed strategy, a control experiment was achieved using bare MnO_2_ QDs without the addition of capping cysteine. It was also found that the fluorescence intensity did not significantly affect the presence of copper, confirming that the presence of the sulfhydryl group of cysteine on the exterior of QDs selectively binds with copper (Supplementary Figure S7).

The reaction mechanism was also studied using the Stern–Volmer equation as Eq. 3:

3 $${F}_{0}/F=1+{K}_{\text{sv}} [Q]$$

where *K*_SV_ is the Stern–Volmer constant, [*Q*] is the molar concentration of copper, and *F* and *F*_0_ are the intensity of fluorescence of Cys@MnO2 QDs in the addition and lack of copper, respectively.

**Fig. 2:**
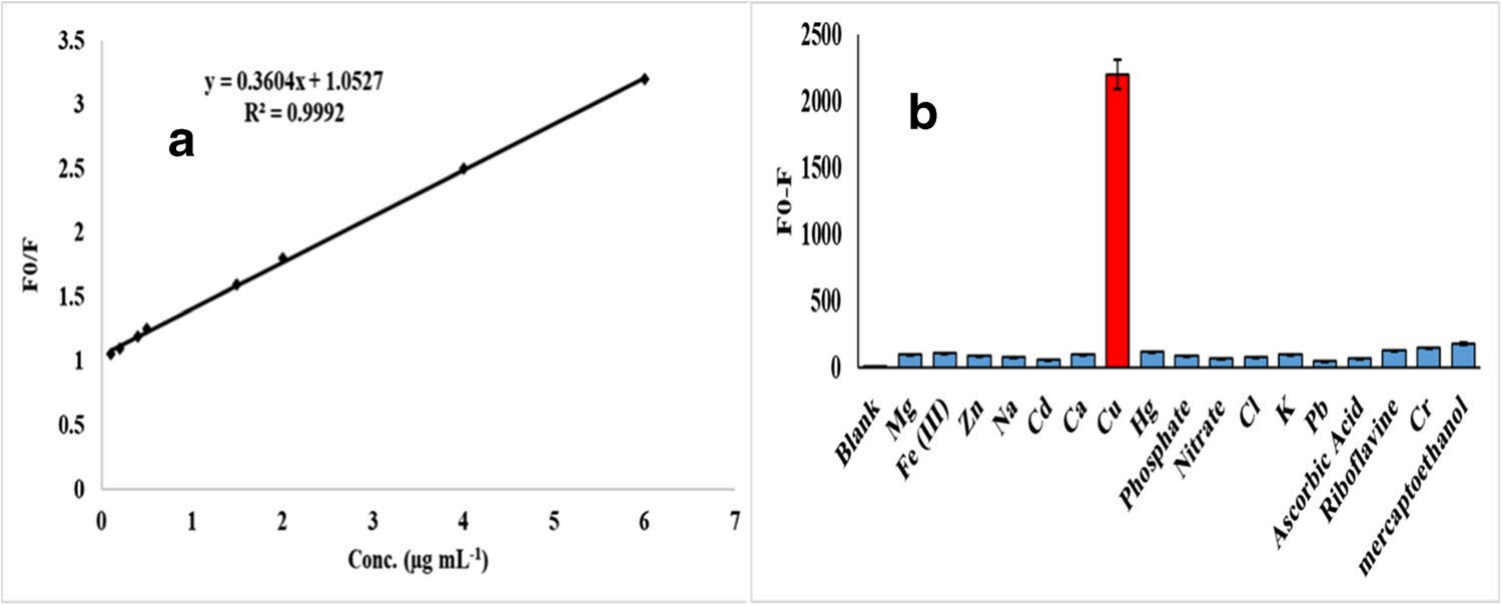
**a** Stern–Volmer equation and **b** the selectivity of the proposed fluorometric method for Cu^2+^ detection using Cys@ MnO_2_ QDs as a probe at the selected optimum conditions. The concentration of all species is 2.0 µg/mL, in the presence of Na_2_S_2_O_5_ as a masking agent for Hg^2+^

The linearity of the Stern–Volmer plot is a clear indication of the dynamic quenching mechanism; copper interacts with the Cys@MnO_2_ QDs resulting in the dynamic quenching mechanism. This process is exactly described by the Stern–Volmer model (Fig. 2a).

### The selectivity of the proposed method

In the cited commercial samples, a few common species were examined for their impacts on the fluorescence emission of Cys@MnO_2_ QDs under ideal circumstances. Ca^2+^, Fe^3+^, Mg^2+^, Na^+^, K^+^, Zn^2+^, Cd^2+^, Hg^2+^, Pb^2+^, NO_3_^−^, PO_4_^3−^, Cl^−^, Cr^3+^, ascorbic acid, 2-mercaptoethanol, and riboflavin are some of the common interferents which were prepared separately at concentrations of 2.0 µg mL^−1^. Then, an amount of 2.0 µg mL^−1^ of Cu was added, and the Cys@MnO_2_ QD fluorescence intensities were measured. The fluorescence intensities remained totally unaltered in the existence of the other interferents, indicating the good selectivity of the proposed protocol toward Cu^2+^ estimation in presence of those common interferents. Regarding Hg^2+^, it was reported that cysteine could form Hg–S bonds through the strong binding preference of cysteine toward Hg^2+^. Prerequisite removal of Hg^2+^ from the commercial samples by chemical complexation with 2% sodium metabisulfite (Na_2_S_2_O_5_) as a selective masking agent may be considered as an effective tool to get the selective detection of Cu^2+^ in the presence of Hg^2+^. Besides, Hg^2+^ in the cited commercial samples is often very low [33]. Figure 2b shows that there was a high selectivity to copper when compared to other interferences.

### Optimization of the creative approach

**Fig. 3:**
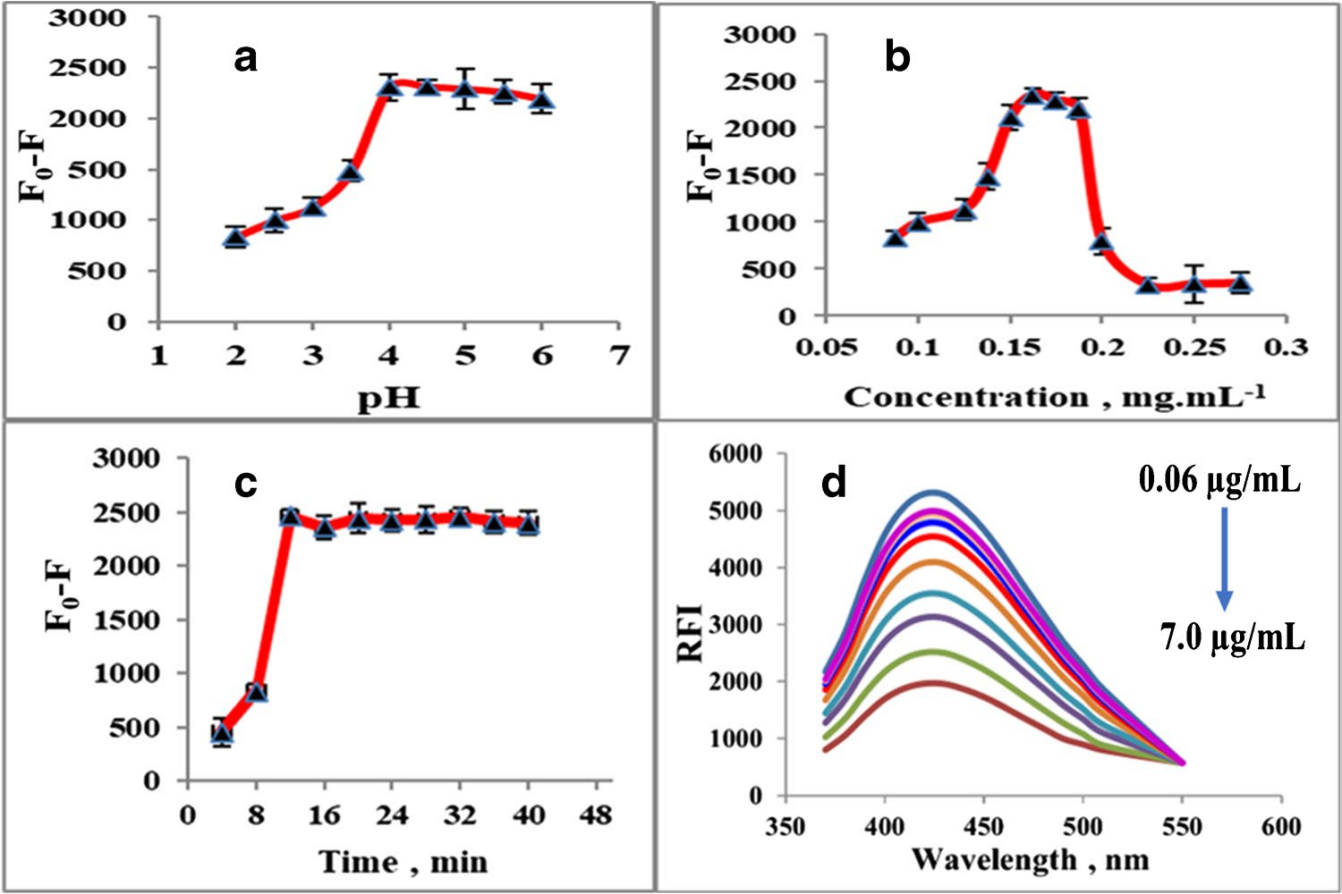
Optimization of the detection of Cu using Cys@MnO_2_ QDs: **a** sample pH value; **b** concentration of Cys@MnO_2_ QDs, **c** standing time, and **d** calibration plot of RFI against the Cu concentration

Several parameters were optimized including sample pH value, Cys@MnO_2_ QD concentrations, and the standing time.pH effect on the RFI of Cys@MnO_2_ QDs in the presence of copper is essential that greatly influences the quenching efficacy of Cys@MnO_2_ QDs by Cu ion. The effect of pH was investigated in the range of 2 to 6. It was observed that at a pH more than 7, the precipitation and formation of Cu(OH)_2_ will affect the experimental results. Besides, Cu becomes no more available for Cu–S formation [34]. It is seen in Fig. 3a that the fluorescence quenching efficiency increases as the pH was increased from 2 to 4. The quenching capacity reaches the maximum at pH 4 and is then kept steady with a small decrease at pH 5 and 6; thus, pH at 4.5 was selected as the optimum pH (Fig. 3a).

Cys@MnO_2_ QDs’ concentration effect on the fluorescence quenching is shown in Fig. 3b. The fluorescence quenching increased with an increase in their concentration up to 0.15–0.18 mg mL^−1^. However, at higher concentrations (above 0.18 mg mL^−1^), a marked decrease in fluorescence of Cys@MnO_2_ QDs occurred.

Also, the effect of reaction time on the fluorescence response of the system was studied (Fig. 3c). The fluorescence quenching became slow until reaching a steady state at 12 min. A further increase in time did not lead to any further noticeable change. Therefore, 12 min was selected as the optimal standing time.

### Analysis performance of copper using Cys@MnO_2_ QDs

In accordance with ICH recommendations, the suggested approach was validated [35]. The emission spectra were performed under the chosen ideal conditions with the incorporation of different copper concentrations (0.06–7.0 µg mL^−1^) as in Fig. 3d. As the concentration of Cu increased, the fluorescence intensity decreased. The creation of a stable Cu–S bond with Cys@MnO_2_ QDs was said to be responsible for the outcomes. The calibration plot was developed by comparing diverse concentrations of Cu to a blank sample. Regression data including linearity range, determination coefficient (*R*^2^), the limits of detection (LOD), and the limits of quantitation (LOQ) were calculated and presented in Table 1.

**Table 1:** Regression parameters for the proposed method for copper quantification

Parameter	Value
Range of linearity (μg mL^−1^)	0.06–7.00
Stern–Volmer equation	*y* = 0.36*x* + 1.05
LOD (ng mL^−1^)	10.97
LOQ (ng mL^−1^)	33.33
Determination coefficient (*R*^2^)	0.999
SD of intercept	1.20

The Stern–Volmer equation [36] was used to explore the mechanism of quenching (Eq. 4).

4 $$\frac{{F}_{0}}{F}=1+ {K}_{\mathrm{SV}} \left[Q\right]$$

where *K*_SV_ is the Stern–Volmer constant, [*Q*] is the molar concentration of copper, and *F* and *F*_0_ are the intensity of fluorescence of Cys@MnO2 QDs in the addition and lack of copper, respectively.

The Stern–Volmer plot, constructed for the ratio *F*_0_/*F* against the Cu concentration, gave a straight line (Fig. 2a). The plot parameters indicate the formation of a quite stable dynamic quenching interaction between Cu and the Cys@MnO_2_ QD shell. The limits of detection were calculated as the following: LOD = 3.3*σ*/*S* and LOQ = 10*σ*/*S*, where *σ* is the standard deviation and *S* is the slope of the regression line.

The accuracy and precision were calculated in terms of percentage recoveries for using Cys@MnO_2_ QDs in Cu^2+^ determination within the concentration of the calibration range, and the results were compared with standard reference copper samples. The results (Table 2) indicate very excellent recoveries for the precision and accuracy parameters. The LOD and LOQ results (Table 1) also confirmed the high sensitivity and suitability of the proposed method.

**Table 2:** Accuracy and precision results of the proposed method for determination of Cu^2+^ comparing with copper standard reference

Sample number	Copper samples		Copper standard reference	
	Taken (µg mL^−1^)	% Recovery * ± RSD	Taken (µg mL^−1^)	% Recovery * ± RSD
1	0.1	100.10 ± 0.58	0.1	100.20 ± 0.21
2	0.5	101.25 ± 1.32	0.5	101.33 ± 0.38
3	1.0	101.24 ± 0.71	1.0	101.60 ± 0.11
4	2.0	99.06 ± 0.44	2.0	100.06 ± 0.90
5	5.0	100.65 ± 0.77	5.0	101.05 ± 0.35
Intra-day precision	1.0	100.80 ± 0.33	1.0	101.10 ± 0.59
	2.0	101.42 ± 0.46	2.0	101.65 ± 0.75
	5.0	100.32 ± 0.88	5.0	100.82 ± 0.65
Inter-day precision	1.0	99.44 ± 0.42	1.0	100.50 ± 0.90
	2.0	99.80 ± 0.97	2.0	100.71 ± 0.27
	5.0	99.69 ± 0.21	5.0	100.19 ± 0.40

*RSD* relative standard deviation

^*^Average of three determinations

**Fig. 4:**
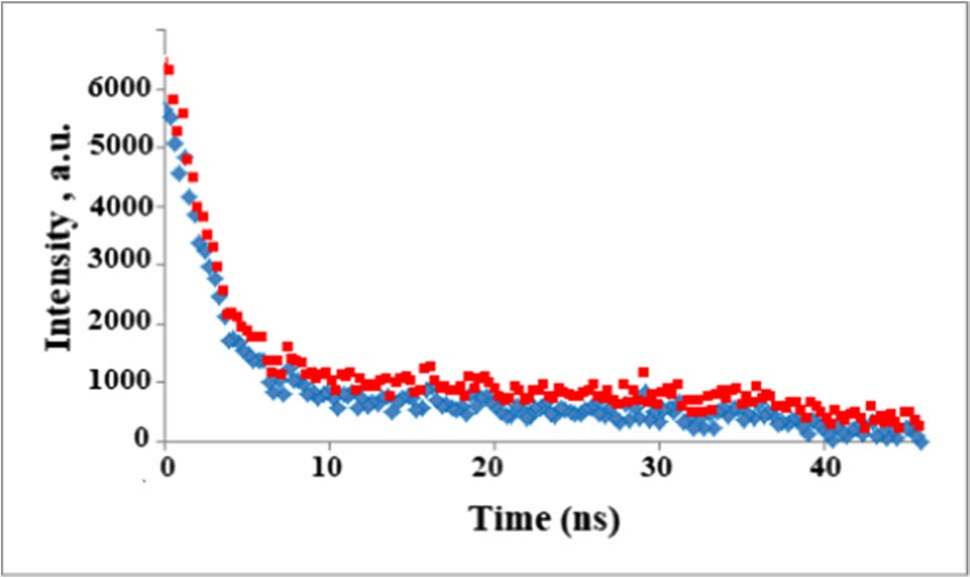
Fluorescence lifetime study of Cys@ MnO_2_ QDs in the absence of Cu (red) and the presence of Cu (blue)

The lifetime decay of Cys@MnO_2_ QDs in the absence and the presence of Cu was also scanned to investigate the mechanism of the quenching magnitude. In addition, the time decay was found to be 5.7 ns as seen in Fig. 4.

### The time stability of Cys@ MnO_2_ QDs

From an applicability point of view, the stability prospect of the probe has a serious magnitude. The more stable a probe is, the greater its viability for a wider application. Thence, the stability over time for the Cys@MnO_2_ QDs was inspected along ordinary conditions of storage. The material was centrifuged after storage for 1 month, washed, and dried at 60 °C for 2 h. Then, the absorbance response of the dispersed solution was measured and compared to that of the freshly synthesized Cys@MnO_2_ QDs. Supplementary Figure S8 shows that the absorbance signal of the stored product was lightly diminished and there was no visible alteration in color after 1 month of storage.

### Method applications

Since copper is very essential for organisms and plays a critical role in numerous biochemical processes and is necessary for enzyme actions, its deficiency could result in conditions such as decreased bone marrow density and osteoporosis [1]. Meanwhile, overexposure to copper is common from excessive use of supplements as well as environmental exposure from daily live activities. Copper toxicity could result in impacted functions of the brain, infertility, and/or insomnia [37]. Therefore, the proposed method was applied for the determination of copper in food samples of chicken meat, turkey, and canned fish. Being also of great advantage in assessing human exposure to heavy metal contaminants, hair samples were also investigated as biological samples for the proposed method [37]. Copper was estimated by the recommended procedure using Cys@MnO_2_ QDs under the selected conditions which agree with those of other reported methods [22]. As shown in Table 3, the results refer to the simplicity of application of the proposed method in quality control during food manufacturing to improve the quality of food and public health improvement.

**Table 3:** Estimation of copper in various types of food and hair using the proposed method

Sample no	Chicken meat	Turkey	Canned fish	Hair
	µg g^−1^*	µg g^−1^*	µg g^−1^*	µg g^−1^*
1	1.33 ± 0.09	1.35 ± 0.08	0.50 ± 0.04	3.34 ± 0.10
2	1.28 ± 0.10	1.29 ± 0.09	0.52 ± 0.03	3.40 ± 0.25
3	1.32 ± 0.08	1.28 ± 0.10	0.48 ± 0.04	3.29 ± 0.18
4	1.30 ± 0.04	1.37 ± 0.07	0.49 ± 0.05	3.34 ± 0.16
5	1.34 ± 0.11	1.33 ± 0.10	0.48 ± 0.07	3.54 ± 0.29

^*^Mean of five determinations

### Comparison study between the proposed method and other reported methods

The parameters of the presented study were compared to those of some previously reported methods [7, 22, 38, 39]. The technique used, range of quantification, and LODs were compared (Table 4). The proposed method overperforms those methods under comparison concerning applicability and sensitivity in terms of LOD and linearity range for quantification [7, 22, 38, 39]. The proposed method was ultrasensitive with higher reliability than other reported methods with an LOD equal to 14.20 ng mL^−1^. The RSDs of the results (Table 4) refer to the better repeatability for applying the proposed method in quality control during food manufacture for improving food quality with a high sensitivity method. In addition, the proposed method is considered environmentally friendly compared to reported methods [8] which are based on using organic solvents and sophisticated instruments [7, 22, 38, 39]. The presented work was established for a green, environmentally friendly, selective, and ultrasensitive method for the estimation of Cu using Cys@MnO_2_ QDs. The sensitivity of Cys@MnO_2_ QDs was also investigated for the assessment of copper ions and their selectivity in the prevention of the contamination of different kinds of food with impurities.

**Table 4:** Comparison between reported methods and proposed nanoprobe for copper

Technique	Linearity range (μg mL^−1^)	LOD (μg mL^−1^)	RSD %	Ref
Atomic absorption spectrometry	4.0–8.0	1.03	2.9	[22]
Electrochemical	90–7000	23.9	3.1	[38]
Fluorescence	0.28–10	0.09	2.1	[39]
spectrophotometry	0.6–15	0.19	2.7	[8]
Fluorescence	0.06–6.0	0.01	1.01	Proposed work

## Conclusion

The proposed study describes a successful synthesis of luminescent Cys@MnO2 QDs as novel nanoprobes via an easy and straightforward ultrasonic method. These nanoprobes were synthesized using MnO_2_ nanosheets as a precursor and cysteine as an exfoliating and protective agent, without the use of co-reactants or modeling, nor the need for expensive or demanding equipment, ensuring a lower cost of production. Although most N-doped QDs exhibited fluorescence that is largely localized in regions of green or blue luminescence, where their physical and chemical properties are lacking [9, 40–42], the synthesized Cys@MnO_2_ QDs gave noticeably improved fluorescence with a high quantum yield compared to the other previously reported methods. Therefore, the photoluminescent MnO_2_ QDs, which were created here using a simple ultrasonic process, are considered a more attractive approach for estimating Cu. The addition of copper leads to the development of a Cu–S bond with the green synthesized quantum dots, where quenching occurs proportionally. The novel fluorescent nanomaterials were found suitable for Cu detection at low concentrations, which is ideal for screening in biological and environmental purposes at a simple operability and low cost. The fabricated luminescent Cys@MnO_2_ QDs have been successfully used for smart estimation of copper detection not only in aqueous solutions but also in food samples and hair. This could signal their promising future and effective applicability in quality control of trace Cu contaminants in other matrices for safety, anticounterfeiting, and legislative purposes as well.

### Supplementary Information

Below is the link to the electronic supplementary material.

Supplementary file1 (DOCX 963 kb)

## Data Availability

All data are available from the corresponding author upon request.
