# Correction to: Development of cysteine‑doped MnO2 quantum dots for spectrofluorimetric estimation of copper: applications in different matrices

**DOI:** 10.1007/s00216-024-05291-z

**Published:** 2024-04-23

**Authors:** Baher I. Salman, Ahmed I. Hassan, Roshdy E. Saraya, Adel Ehab Ibrahim, Bassam Shaaban Mohammed, Hany A. Batakoushy, Sami El Deeb, Yasser F. Hassan

**Affiliations:** 1 Pharmaceutical Analytical Chemistry Department, Faculty of Pharmacy, Al-Azhar University, Assiut Branch, Assiut, 71524 Egypt; 2 Pharmaceutical Analytical Chemistry Department, Faculty of Pharmacy, Port Said University, Port Said, 42511 Egypt; 3 Natural and Medical Sciences Research Center, University of Nizwa, Birkat Al Mauz, P.O. Box 33, Nizwa, 616 Sultanate of Oman; 4 Department of Pharmaceutical Analytical Chemistry, Faculty of Pharmacy, Menoufia University, Shibin‑Elkom, 32511 Egypt; 5 Institute of Medicinal and Pharmaceutical Chemistry, Technische Universitaet Braunschweig, 38106 Braunschweig, Germany

**Correction to: Analytical and Bioanalytical Chemistry (2023) 415:5529–5538**

10.1007/s00216-023-04827-z

The original version of this article unfortunately contained a mistake.

After careful review, the authors discovered that there had been a partial mix-up in figure 1. The specific error relates to the TEM image of the developed Cys@MnO_2_ QDs which were displayed as part (a) of Figure 1 (Fig. 1a) in the original article. Unfortunately, during the publication process, the figure was mistakenly replaced with an incorrect version, leading to an inaccurate representation of the study findings. The correct figure 1 has been included below. The authors would also like to point out that based on the proposed error, we should regretfully replace the graphical abstract of the manuscript, which includes the mixed-up image, with the newly presented graphical abstract. Finally, the authors regret the non-intentional error and confirm that this partial mix-up in figure 1 does not affect the integrity or accuracy of the underlying data, analysis, or conclusions presented in the manuscript. The error solely pertains to the partial attachment of the figure and not the content it represents. The data related to the manuscript remain unchanged and have the same integrity as originally presented.

**The old version of figure 1:** 
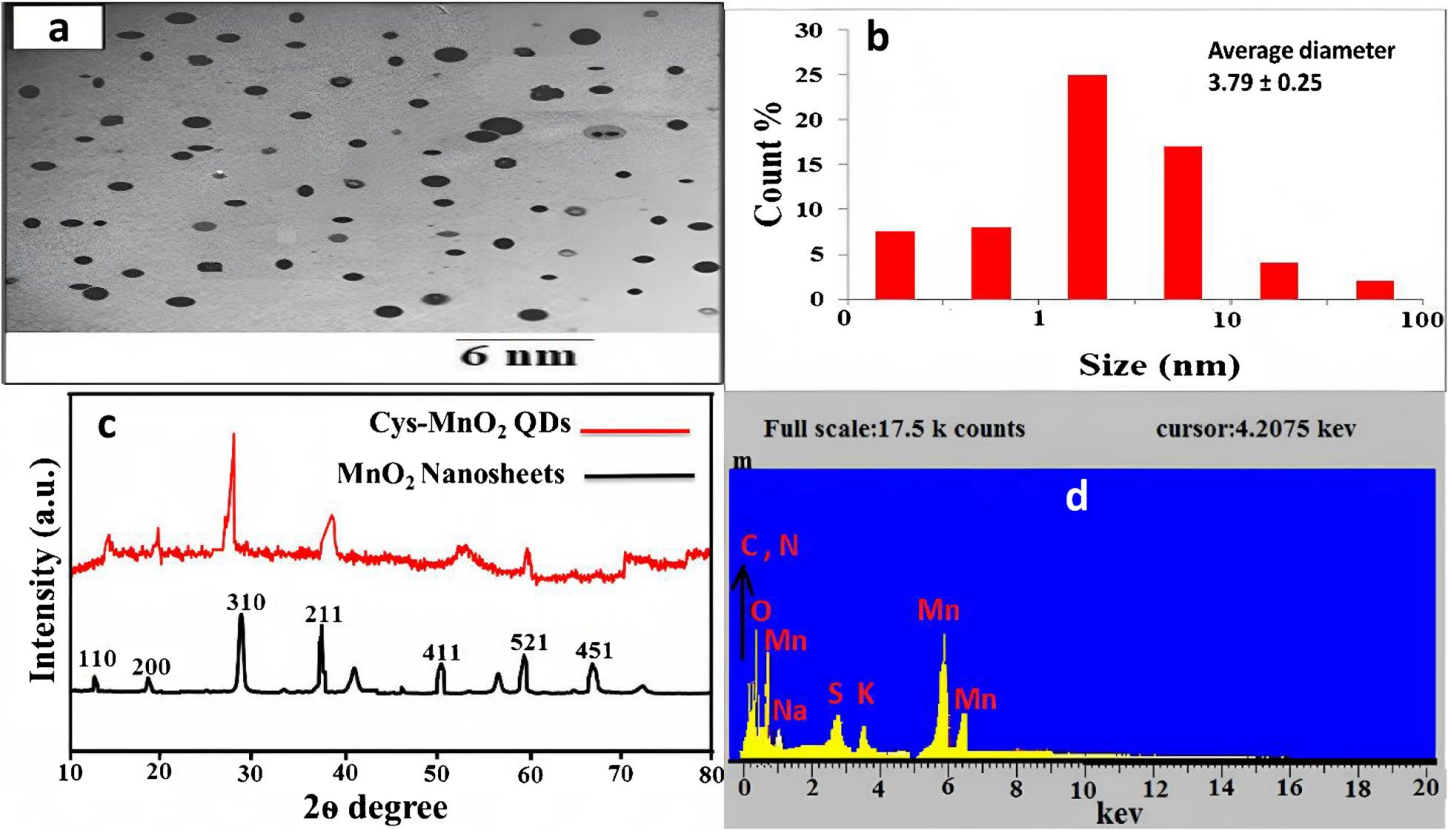


**Figure 1:** TEM images of Cys@MnO_2_QDs (a), DLS of Cys@MnO_2_QDs (b), PXRD (c) and EDX for Cys@MnO_2_QDs (d).

**The correct version of figure 1:** 
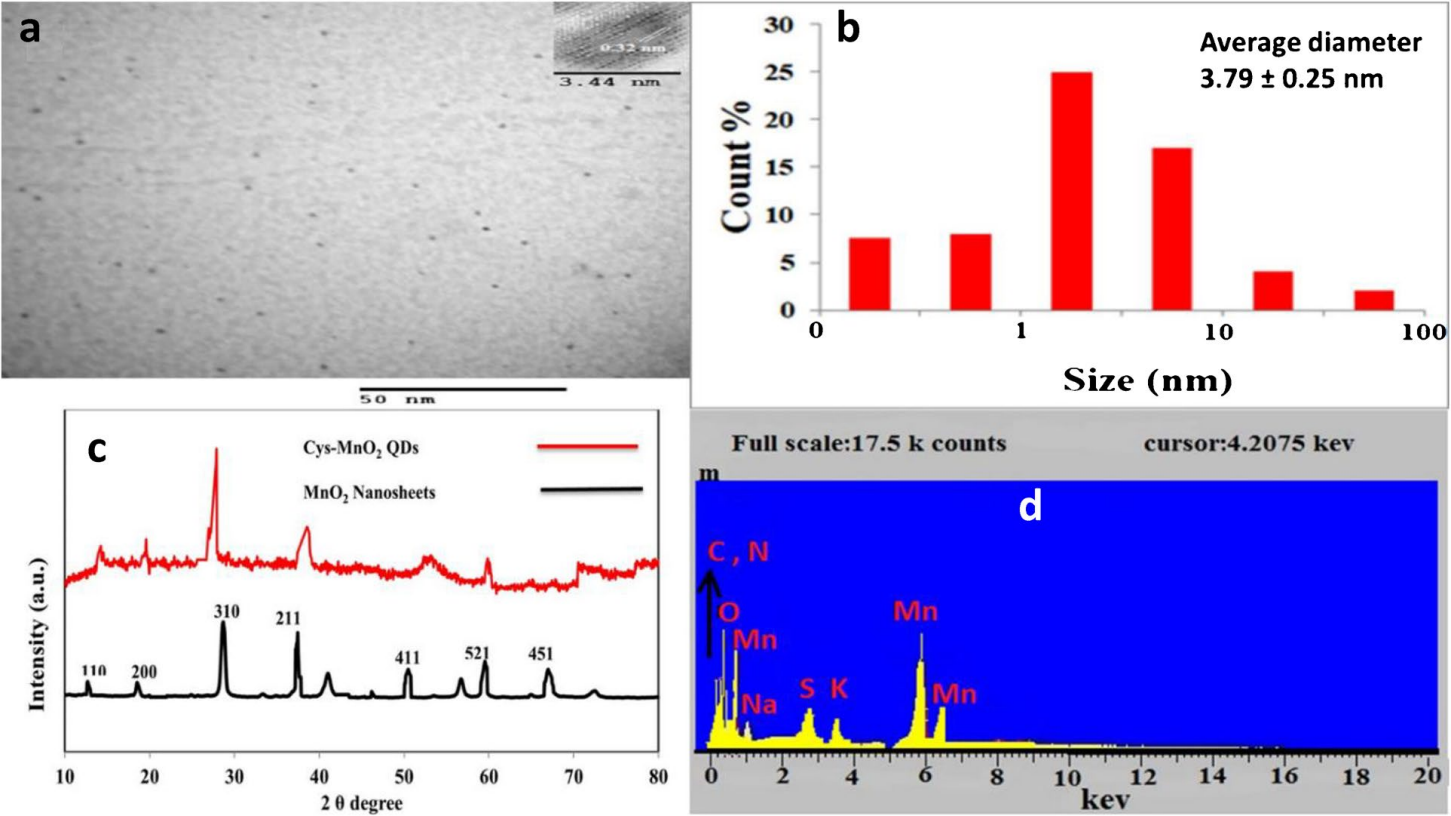


**Figure 1 corrected:** TEM images of Cys@MnO_2_QDs (a), DLS of Cys@MnO_2_QDs (b), PXRD (c) and EDX for Cys@MnO_2_QDs (d).

**The old version of the graphical abstract:** 
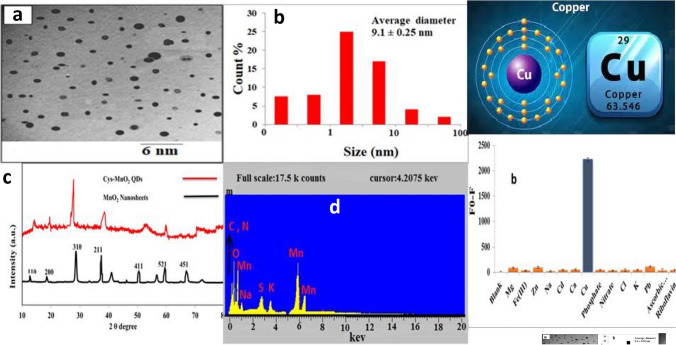


**The correct version of the graphical abstract:** 
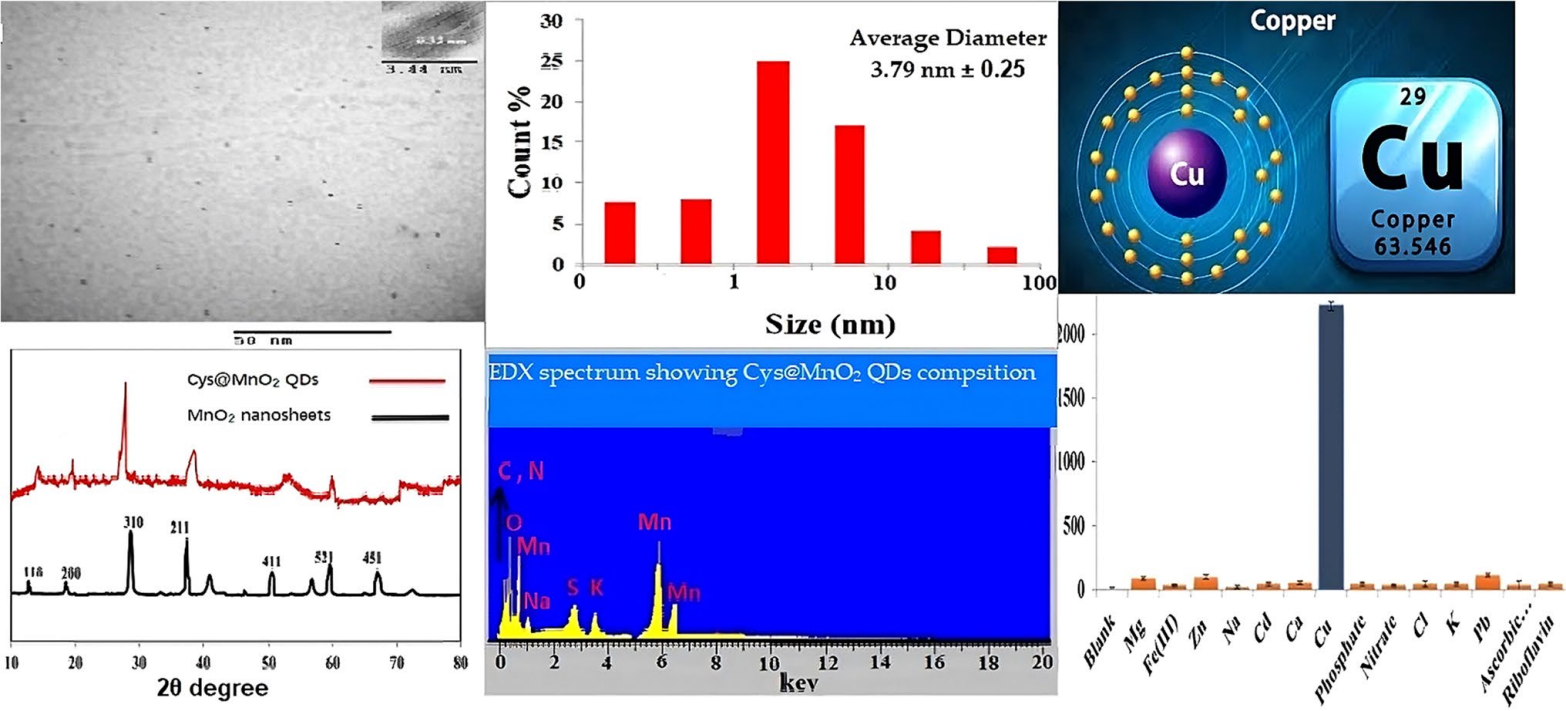


The original article has been corrected.

