# Supplementary material for: Development of cysteine-doped MnO2 quantum dots for spectrofluorimetric estimation of copper: applications in different matrices
Source: Anal Bioanal Chem. 2023 Jul 11;415(22):5529–38. doi: 10.1007/s00216-023-04827-z (PMC10444647; doi:10.1007/s00216-023-04827-z)
Supplement: Supplementary file 1 — Supplementary file1 (DOCX 963 kb) [file 216_2023_4827_MOESM1_ESM.docx]

**Electronic Supporting Materials**

**Development of cysteine-doped MnO_2_ quantum dots for spectrofluorimetric estimation of copper: applications in different matrices**

Baher I. Salman^1^, Ahmed I. Hassan^1^, Roshdy E. Saraya^2^, Adel Ehab Ibrahim^2,3^, Bassam Shaaban Mohammed^4^, Hany A. Batakoushy^4^, Sami El Deeb^5,3,*^, and Yasser F. Hassan^1^

*^1^*  *Pharmaceutical Analytical Chemistry Department, Faculty of Pharmacy, Al-Azhar University, Assiut branch, Assiut 71524, Egypt.*

*^2^*  *Pharmaceutical Analytical Chemistry Department, Faculty of Pharmacy, Port Said University, Port Said 42511, Egypt.*

*^3^*  *Natural and Medical Sciences Research Center, University of Nizwa, P.O. Box 33, Birkat Al Mauz, Nizwa 616, Sultanate of Oman.*

*^4^*  *Department of Pharmaceutical Analytical Chemistry, Faculty of Pharmacy, Menoufia University, Shibin Elkom, 32511, Egypt.*

***^5^*** *Institute of Medicinal and Pharmaceutical Chemistry, Technische Universitaet Braunschweig, Germany*

* Corresponding author at: [s.eldeeb@tu-bs.de](mailto:s.eldeeb@tu-bs.de) (S.E.)


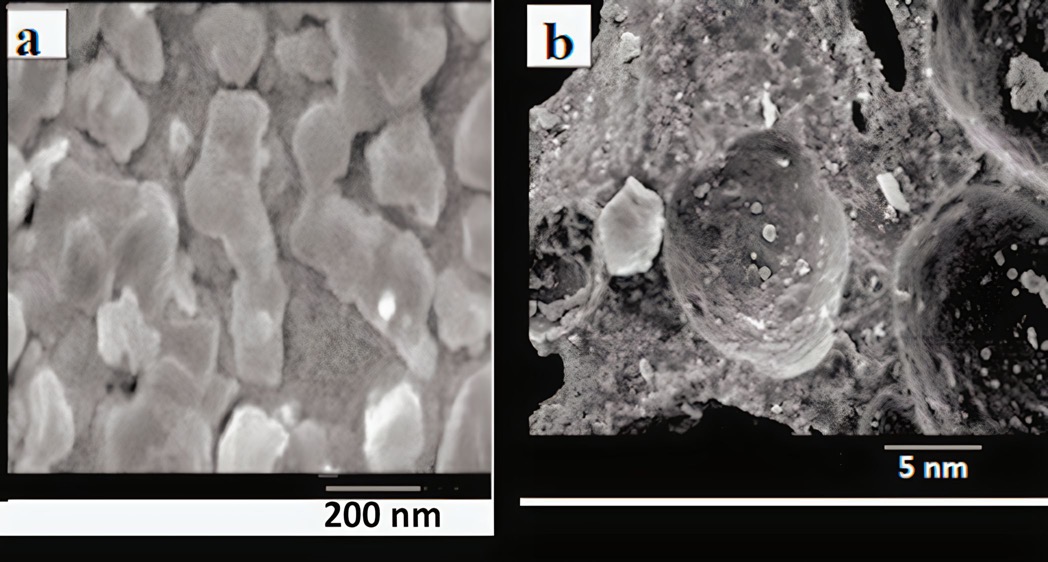


**Figure S1:** SEM image a) MnO_2_ nanosheet and b) Cys-MnO_2_ QDs.


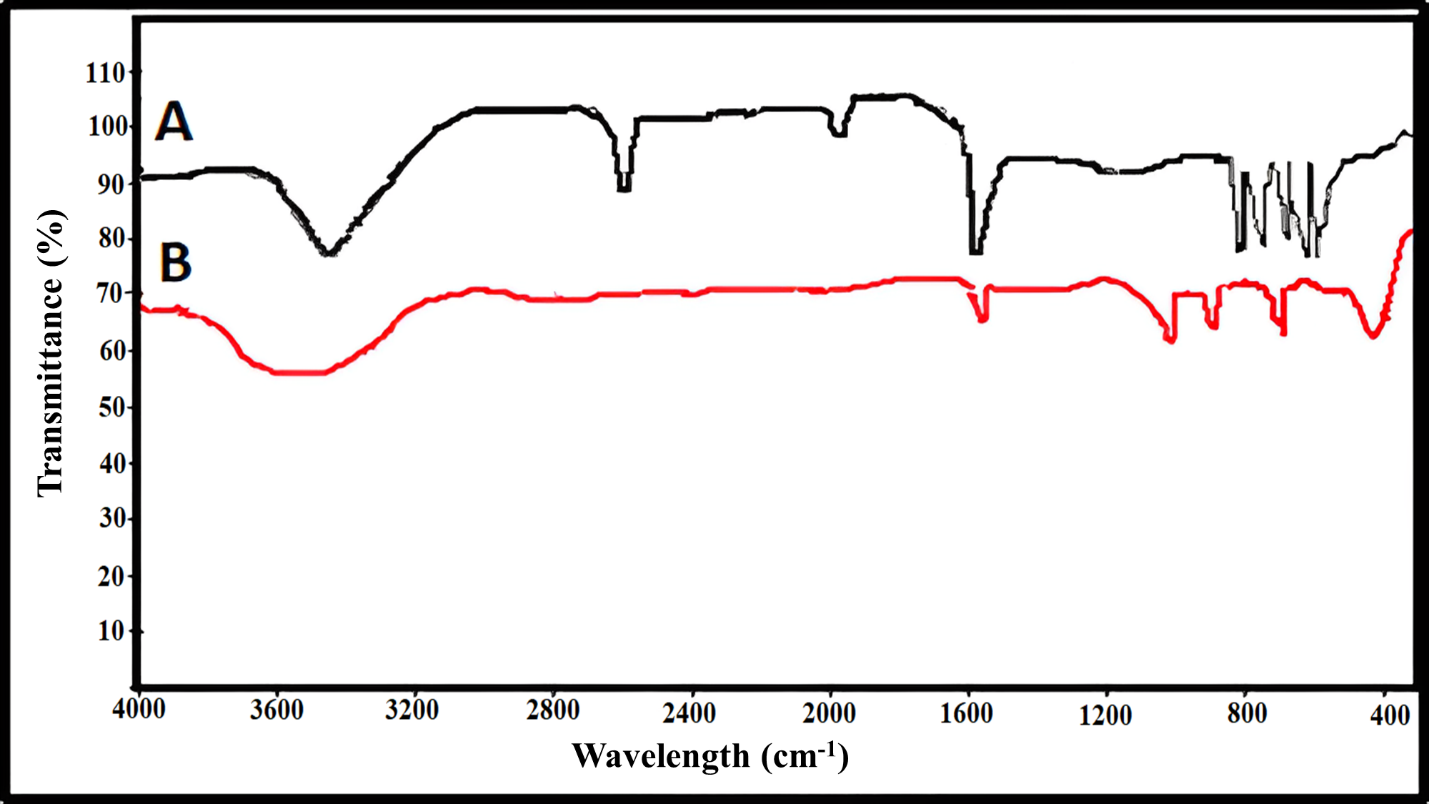


**Figure S2:** FTIR spectra of (A) pure Cysteine (B) Cys-MnO_2_ QDs


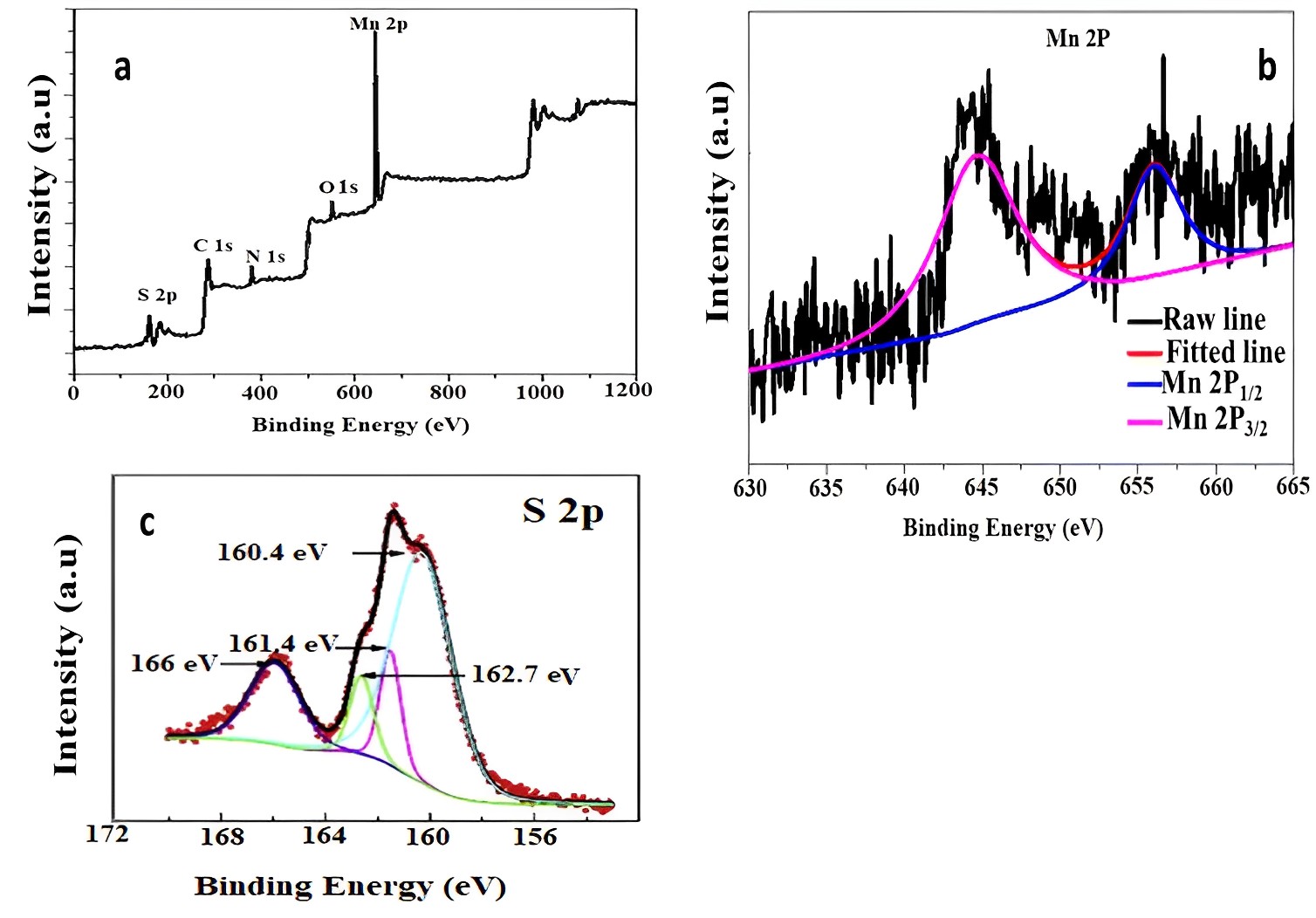


**Figure S3**: **a)** XPS for Cys-MnO_2_ QDs, **b)** Mn spectra and **c)** S spectra for Cys-MnO_2_ QDs.


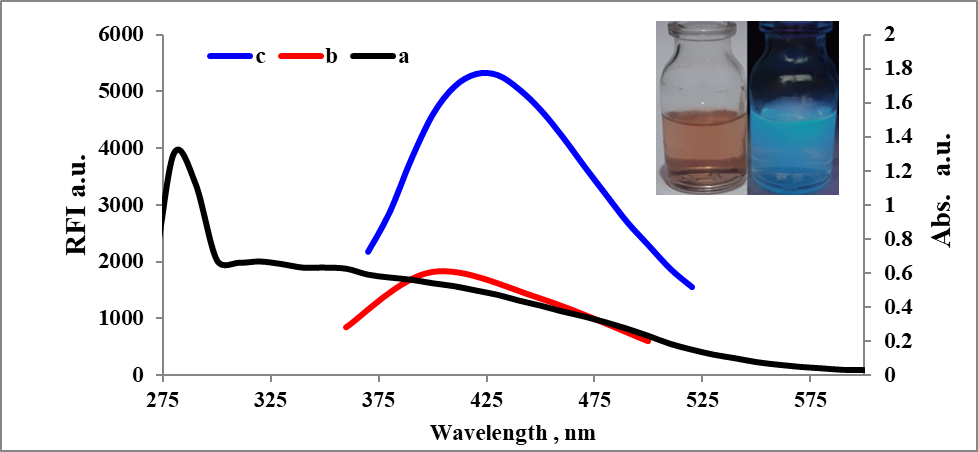


**Figure S4:** Overlay spectra of (a) UV-Vis of Cys-MnO_2_ QDs, (b) emission of MnO_2_ QDs, and (c) emission of Cys-MnO_2_ QDs, inset: Cys-MnO_2_ QDs under daylight (left) and UV light at 365 nm wavelength (right)


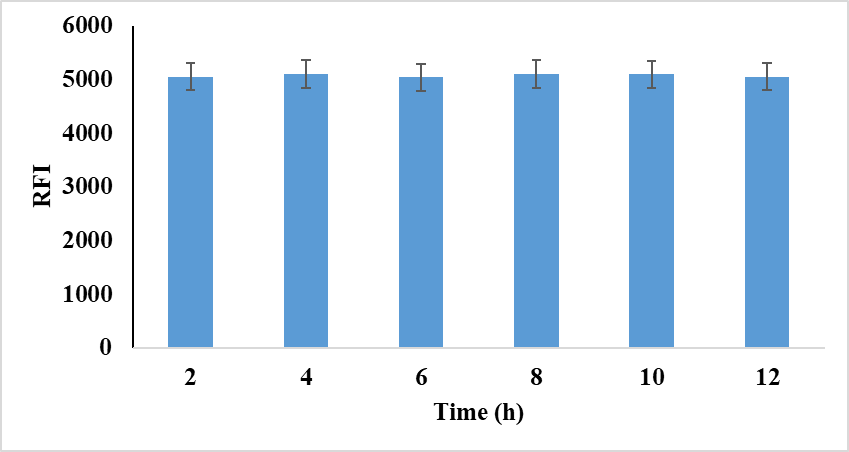


**Figure S5:** Photostability of the obtained Cys-MnO_2_ QDs


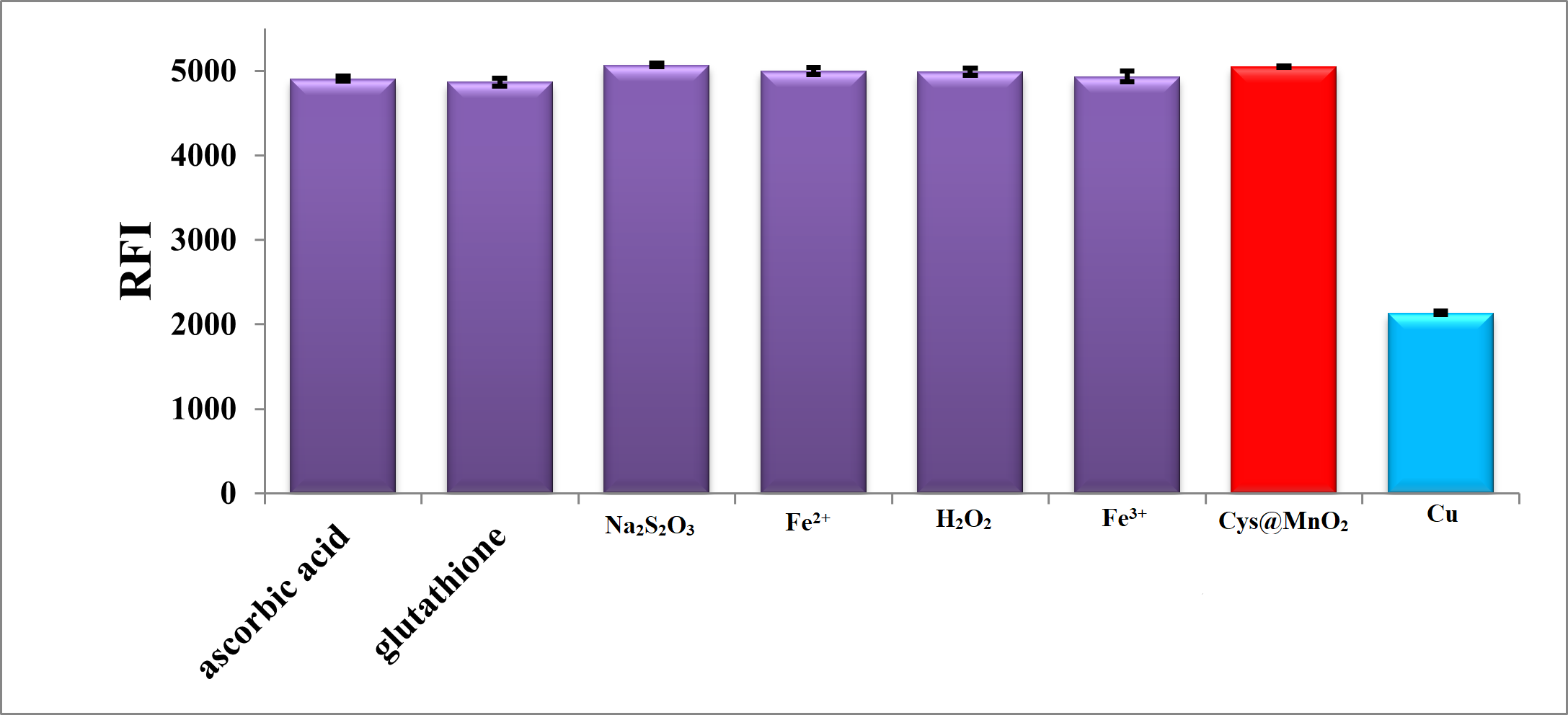


**Figure S6:** Fluorometric response of Cys@MnO_2_ QDs in presence of 2µg mL^-1^ of Cu^2+^ and some oxidants, and some reductants


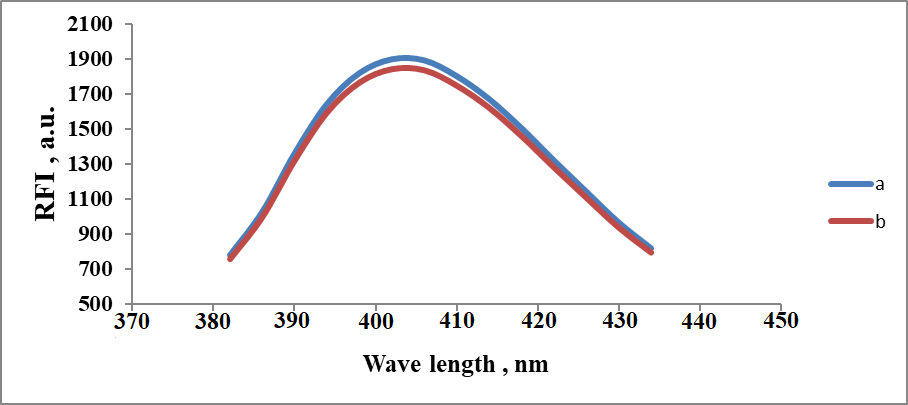


**Figure S7:** The fluorescence signal of the bare MnO_2_ QDs in the absence (a) and presence (b) of 2µg mL^-1^ Cu^2+^.

**Figure S8:** UV-Vis spectra of Cys@MnO_2_ QDs at once (blue), and after 1 month storing (red), inset refers to the color change during 1 month storing
